# Altiplano agricultural origins was a process of economic resilience, not hardship: Isotope chemistry, zooarchaeology, and archaeobotany in the Titicaca Basin, 5.5-3.0 ka

**DOI:** 10.1371/journal.pone.0325626

**Published:** 2025-06-25

**Authors:** Luis Flores-Blanco, Morgan Hall, Luisa Hinostroza, Jelmer Eerkens, Mark Aldenderfer, Randall Haas

**Affiliations:** 1 Department of Anthropology, University of California, Davis, California, United States of America; 2 School of Human Evolution and Social Change, Arizona State University, Tempe, Arizona, United States of America; 3 Department of Ethnobotany and Economic Botany of the Museum of Natural History, UNMSM, Lima, Peru; 4 Department of Anthropology and Heritage Studies, University of California, Merced, California, United States of America; 5 Department of Anthropology, University of Wyoming, Laramie, Wyoming, United States of America; Sapienza University of Rome: Universita degli Studi di Roma La Sapienza, ITALY

## Abstract

Prevailing models of agricultural origins tend to envision that economic hardship drove the transition from foraging to farming economies. Growing human populations and the depletion of high-ranked animal resources forced humans into increasingly intensive and dependent relationships with plant foods. Current evidence from the Andean *Altiplano* (High Plateau, 3800 masl) identifies the Terminal Archaic Period (5.0–3.5 cal. ka) as the period of economic transition from Archaic foraging economies to Formative Period agro-pastoral economies. Consistent with models of agricultural origins, isotope chemistry (δ^13^C_collagen_, δ^13^C_apatite_, δ^15^N_collagen_) of human bone samples from 16 individuals from the Terminal Archaic sites of Kaillachuro and Jiskairumoko (5.3–3.0 cal. ka) indicates that C_3_ plants comprised approximately 84% of the dietary protein. Archaeobotanical data show that chenopods may have been the most important subsistence resource, and zooarchaeological remains indicate that protein was derived from camelid meat. Inconsistent with the working model of plant intensification, the Terminal Archaic diets reported here are statistically indistinguishable from previously published values of Early—Late Archaic (9.0–6.5 cal. ka) individuals in the same region, which also show approximately 84% of protein coming from plants. Rather than being a process of dramatic dietary change and economic hardship, the agricultural transition on the Altiplano appears to have been one of remarkable resilience in which plant:meat ratios remained relatively stable over six millennia, spanning the transition from Archaic foraging and hunting to Formative farming and herding economies. Plant and animal domestication on the Altiplano thus represents a process of economic sustainability rather than one of food insecurity and hardship, as many prevalent agricultural origins models would suggest.

## Introduction

The transition from foraging to food-producing economies is one of the most consequential transitions in human sociocultural evolution. While early views characterized this transition as one of monotonic improvement in human livelihoods, subsequent and currently prevailing views envision a non-monotonic process in which the transition to agricultural economies are seen as a process of economic downturn and hardship wrought by demographic circumscription, tragedy of the commons, and food shortage [1–7]. In this latter view, human forager economies relied on diverse subsistence resources including preferred, high-ranked animal foods. As increasing human populations reduced access to preferred resources, diet-breadth expansion resulted in the incorporation of lower-ranked plant and animal foods and their intensification [8–12]. Beyond these economic factors, some researchers also emphasize that the transition was influenced by ideological and symbolic factors, such as surplus production for ceremonial and social exchange as a means of social competition [13], cognitive shifts in human societies [14], and environmentally driven [15,16].

This significant economic transition occurred in the Central Andes during the Terminal Archaic Period between 5.3–3.5 cal. ka [17,18,19,20,21–23,24]. Current subsistence data from the Middle Archaic Period to the Early Formative Period (8.0–3.0 cal ka) indicate that a mixture of plants and animals characterized the subsistence economies of Andean Altiplano inhabitants [18,25,21–23,26–28]. Archaeobotanical research reveals the early cultivation of quinoa (*Chenopodium quinoa*), wild chenopods, and other taxa during the Terminal Archaic (5.3–3.5 cal ka) and Early Formative (3.5–3.0 cal ka) periods [26,21,29–31]. The earliest evidence of quinoa is from the Jiskairumoko site [21], where at least 38 quinoa seeds show traits comparable to domestic forms, such as large size and a thin testa (<20 μm). These seeds were collected at Jiskairumoko’s Burial 3 and Pithouse 3, which date to the Terminal Archaic Period, as well as Rectangular Structures 1 and 2 from the Early Formative Period. Quinoa is also the primary plant species recovered from the Chiripa site, both cultivated and wild. Researchers assume that early cultivators grew both types of quinoa in their gardens rather than farm fields [26].

Wild tubers appear to have been a staple food among the inhabitants of the Titicaca Basin since at least the Middle Archaic Period [28]. Early intensive consumption of tubers—ostensibly potatoes (*Solanum tuberosum*)—is suggested by the presence of charred parenchyma tissue, dental-wear patterns, and human-bone isotope chemistry at the Middle/Late Archaic site of Soro Mik’aya Patjxa [27,28]. At the Terminal Archaic Period site of Jiskairumoko, potato starch grains were discovered on fourteen groundstone artifacts, suggesting intensive potato use and potential domestication at that time [23,24]. Additionally, potato and other tuber starch grains, including varieties specific to potato and oca (*Oxalis tuberosa*), were discovered within the strata of the Early Formative site Chiripa [32,33]. Genetic evidence furthermore reveals that biological adaptations for increased human starch digestion appeared approximately 8 cal. ka, consistent with models of early intensive tuber consumption [34,35].

Lithic assemblages also inform on past subsistence practices across the farming transition. Excavations at Soro Mik’aya Patjxa show that informal groundstone was commonly used 8.0–6.5 cal. ka [36]. More formal groundstone artifacts were recovered from Jiskairumoko, with a notable increase from the Terminal Archaic to the Early Formative periods [23,24]. Grinding stones are also recorded at the Early Formative Period sites of Chiripa and Camata, with seemingly little innovation in technology over many centuries [37,38], indicating that plant processing remained a consistent and important activity.

Among the evidence for plant-food consumption, highland archaeological sites also yield an abundance of projectile points, suggesting that hunting remained a significant economic undertaking throughout the Archaic and Early Formative periods [37,39–42]. Whereas dart points dominate Archaic assemblages prior to 5 cal. ka, small triangular points—thought to be arrowheads—became abundant during the Terminal Archaic Period [18,43]. Large-mammal bone dominates Archaic faunal assemblages in the northern Titicaca region [22]. In the more arid southern Titicaca Basin, Archaic and Formative assemblages reveal a greater variety of prey remains, including large mammals such as camelids and cervids, but also significant numbers of small mammals such as rodents, vizcacha, birds, and fish [40,44–49].

Despite efforts to reconstruct subsistence practices across the transition to agriculture in the Titicaca Basin, a majority of evidence continues to be indirect. The prevalence of projectile points and faunal remains and the paucity of charred plant remains could reflect meat-focused economies or preservation bias [27,32,33]. Even among plant foods, quinoa seeds are more likely to preserve than tuber remains. New data sources are necessary to evaluate competing hypotheses about early diets and dietary change in the Titicaca region [23,24,32,33].

The stable isotope chemistry of human bone collagen and bioapatite offer effective ways to directly estimate broad patterns in human diets [20,27,50–53]. This approach is currently under-utilized on the Terminal Archaic and Early Formative assemblages in the Titicaca Basin [25,54,55]. To date, three investigations have used stable isotopes for these periods, primarily focusing on the Formative Period or later. However, these studies are hampered by small sample sizes and problems of chronological control. Based on projectile points, the archaeological site of Muruqullu is the single Terminal Archaic case study. That study provided stable isotope values from seven individuals. High δ^15^N and low δ^13^C values suggest the consumption of large amounts of lake resources and terrestrial mammals [55]. However, the qualitative methods used for dietary reconstruction and lack of radiocarbon dates limit interpretation [56].

Other stable isotope studies of human remains from the Andean Altiplano demonstrate that Terminal Archaic and Early Formative populations depended primarily on terrestrial resources such as C_3_ plants, most likely potatoes and quinoa, as well as meat from terrestrial animals such as camelids and small fauna [25,54,55]. Accordingly, most previous studies on human isotope chemistry focus on foragers before 5 cal. ka and agropastoral populations after 3 cal. ka, but values on humans during the period of economic transition remain sparse and ambiguous.

Reconstructing the dietary foundations that sustained this significant economic transition is crucial to understanding watershed socio-political developments including the establishment of the first villages, the construction of ceremonial burial mounds, and expanding exchange networks that facilitated the introduction of non-local goods like obsidian and technologies such as archery and ceramics [8,17,18,41–43,57–62]. Nevertheless, the nature and tempo of this transition remains unclear.

Given prevailing views of agricultural origins, we hypothesize that the transition to agropastoral economies in the Titicaca region was a gradual, protracted process in which growing, increasingly circumscribed forager communities were forced to expand diets and intensify their relationships with plant foods in way that led to their domestication. Ethnographic studies of recent hunter-gatherer populations show that meat can make up 20–65% of dietary intake, with higher meat consumption expected in colder environments [6,63,64], which typically results in elevated nitrogen isotope values. If similar forager subsistence patterns held in the prehistoric Andean Altiplano, we would expect δ¹⁵N and δ^13^C values to decrease over time as diets shifted toward greater reliance on cultivated plants, as seen in other regions during the Neolithic transition [65–67]. However, this trend could be confounded by the introduction of C_4_ domesticated plants [68]. This scenario, though, should not be expected for the Titicaca Basin during this transitional period, as maize was not introduced until the Middle/Late Formative Period [69]. We would also expect a rise in inter-individual isotopic variance, as people experimented with diverse subsistence strategies. This pattern may reflect cycles of dietary intensification and de-intensification, shaped by ecological or social change, and aligns with ethnographic and archaeological models that describe domestication as a gradual, diverse, multidirectional, discontinuous and reversible process requiring sustained investment [5,70,71].

We should furthermore expect lower δ^15^N and δ^13^C in the Terminal Archaic relative to the preceding Early—Late Archaic values, which previous research shows average 9.4‰ and −18.7‰, respectively [27]. Isotope-based dietary reconstructions for the Terminal Archaic Period should similarly show greater plant contributions compared to the preceding Early—Late Archaic reconstructions, which reveal plant contributions of approximately 80% (60–95%) [27]. During this transitional phase, we also expect greater inter-individual isotopic variance as communities experimented with diverse subsistence strategies, reflecting experimentation and uneven adoption of cultivation and cycles of dietary intensification and fallback. Once agropastoral systems are firmly established, diets become standardized around cultivated staples, leading again to lower variance. Given the premise of increasing engagement with lower-ranked food resources during the Terminal Archaic, we also expect to observe an increasing abundance of small mammals, birds, and fish in faunal assemblages. Finally, archaeobotanical assemblages are expected to show an increasing abundance of taxa that would eventually be domesticated, particularly potatoes and quinoa.

**Fig 1:**
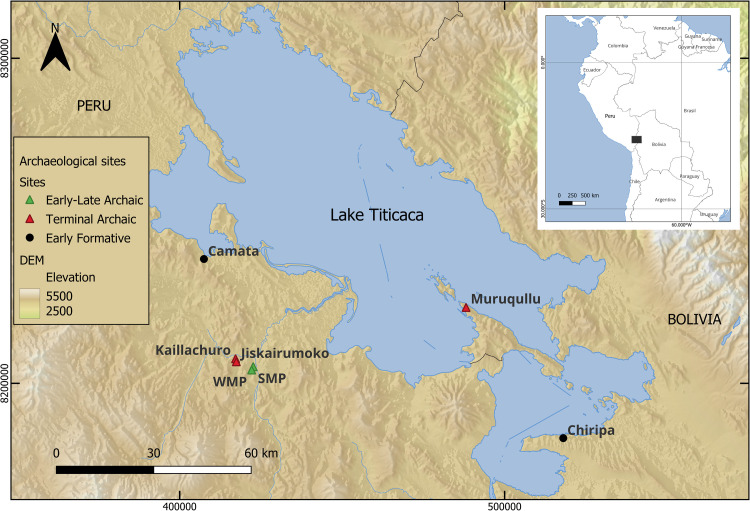
Location of Kaillachuro (KCO) and Jiskairumoko (JKM) and other archaeological sites in the Titicaca Basin, Andes. Early – Late Archaic Period sites (green triangles), Terminal Archaic sites (red triangles) and Early Formative (black dots) mentioned in the text are also indicated on the map. SMP = Soro Mik’aya Patjxa, WMP = Wilamaya Patjxa. Digital elevation model from GMTED2010 [72]. Map created with R and QGIS [73,74]).

Recent excavation of burial mounds at Kaillachuro (KCO) and earlier excavations at the nearby village site of Jiskairumoko (JKM) (Fig 1), sites that span this crucial economic transition in the Titicaca Basin, provide an opportunity to investigate the dietary habits of the region’s first food producers [39,41,75,76]. This study explores the dietary patterns of 16 individuals from these two sites by analyzing stable isotopes of carbon and nitrogen in collagen and carbon in bioapatite, alongside faunal and archaeobotanical remains also recovered from the same sites. Based on dietary reconstructions from preceding Early—Late Archaic periods [27,22,28], as well as comparative archaeological and ecological data on economic conditions during the Terminal Archaic and Early Formative periods of the Andean Altiplano [18,25,20,26,21,23,31,24,33,40,44–49,54,55,77–79], archaeological studies predict a mixed diet, including domesticated plants and animals, among Kaillachuro and Jiskairumoko consumers.

## Materials and methods

At Kaillachuro and Jiskairumoko, excavations revealed human interments in burial mounds and pits, providing new opportunities to evaluate subsistence models at a key moment in the trajectory to social complexity on the Andean Altiplano. Kaillachuro is an early burial mound site that dates between 5.3 and 3.0 cal. ka, based on a sequence of 19 radiocarbon dates, four distinct periods of occupation, temporally-diagnostic projectile points, and ceramic types [41,75]. Less than a kilometer away, Jiskairumoko is a pit-house village with human burials around the houses. These burials are dated by 26 radiocarbon dates that place them between 5.2 and 3.4 cal. ka [39,76]. The Kaillachuro dates mostly consist of direct ^14^C dating of human bone collagen, supported by stratigraphic evidence. Jiskairumoko is dated by a combination of ^14^C dating of charcoal from stratigraphically secure archaeological features and levels.

In short, both sites were used for more than two thousand years, principally in the Terminal Archaic Period, extending into the Early Formative Period. The human bone samples and other macro-organic remains in this study come from both sites. The subsequent sections delineate the analytic methods for human bone isotope chemistry, statistical analysis, zooarchaeological analysis, and archaeobotanical analysis.

### Analysis of stable isotopes in human bone

Collagen (δ^13^C_collagen_ and δ^15^N_collagen_) and bone carbonate (δ^13^C_apatite_) together provide a general picture of the diet of past human remains [50–52,80–82]. The isotopic composition of the foods ingested by animals, including humans, become incorporated into their tissues, including bone. Thus, the isotopic composition of animal tissues broadly reflects the isotopic composition of the foods they consume. The isotopic composition of biological tissues can be characterized using mass spectrometry, lending insights into trophic dynamics. Photosynthesis is the primary mechanism that affects the carbon component of plants, and therefore, the animals that feed on them. Plants are categorized as C_3_, C_4_, and CAM based on their method of photosynthesis [83]. Conversely, the analysis of the stable nitrogen isotopes provides an estimate of trophic position, which refers to the specific place an organism occupies in the food pyramid. Thus, those organisms that mostly consume plants are anticipated to have lower nitrogen isotopic values relative to those that consume more meat [53,80,84].

Twelve human bone samples from the Kaillachuro site predominantly derive from burials within Mound 4, including interments in burial pits, stone cists and a small number of isolated elements from stratified deposits. The majority (72%) of these remains were directly dated using radiocarbon, placing them in the Terminal Archaic Period, while a smaller subset is attributed to the Early Formative Period. The remaining samples were dated based on stratigraphic relationships. Four human bone samples from Jiskairumoko originate from archaeological deposits. Of these, only one was indirectly dated by radiocarbon, as it was part of Burial 3 at Jiskairumoko. Another sample comes from a deposit dated to the Terminal Archaic. The remaining two samples come from uncertain contexts (likely plowed area), and given this uncertainty, we conservatively assign these two samples to the end of the site’s occupation during the Early Formative Period [39] (S3 Data in S1 File).

To extract collagen from bone, we used a modified Longin procedure [85]. Approximately 2 grams of cortical bone was thoroughly cleaned of visible external impurities using a manual drill. Samples were then sonicated in deionized water and left to dry. Of this sample, 1.5-grams were devoted for collagen extraction, while the rest was used for bioapatite analysis. The specimen was subjected to a 24-hour drying process, weighed, and then immersed in a 0.5 M hydrochloric acid (HCl) solution, with HCl replaced every two days, until the sample was fully demineralized. Subsequently, each sample was rinsed with deionized water and submerged in a 0.125 M sodium hydroxide (NaOH) solution for a duration of 24 hours to eliminate humic acids. The samples were subsequently washed once more with deionized water and placed in slightly acidic (pH 3) water in an oven set to 70°C to solubilize the collagenous fraction. The liquid in the vial was subsequently extracted and separated from any leftover particulates using a pipette and transferred to a lyophilizer to remove water, thereby isolating the collagen fraction. Subsequently, 1–2 mg of collagen was weighed into a tin capsule and submitted for isotopic analysis.

For bioapatite samples, the remaining 0.5 g of cleaned and dried bone was powdered in a mortar and pestle. Then, 0.04 g of bone powder was weighed into a labeled vial and treated to remove organic remains. We first added a 1.5% sodium hypochlorite (bleach) solution in the sample vial, for two 8–12-hour treatments, discarding and changing the base solution each time. After rinsing with dH_2_O, the sample was then immersed in 1M acetic acid solution, for two 8-to-12-hour treatments, with the fluid discarded after each treatment. Finally, the sample was again rinsed in dH_2_O and dried.

Bone processing took place in the UC Davis Archaeometry Laboratory. Isotopic analyses were conducted in the UC Davis Stable Isotope Facility and measured for δ^13^C and δ^15^N by EA-IRMS. Powdered samples were analyzed for δ^13^C in the carbonate fraction by GasBench-IRMS. The laboratory calibrated the collagen results with respect to international reference materials, Vienna PeeDee Belemnite (VPDB) for δ^13^C and air for δ^15^N.

Raw isotopic data from human archeological remains are offset from the individual’s diet due to isotopic enrichment within the human body. Previous studies suggest the enrichment for δ^13^C is 4.5–6.0‰, while δ^15^N offsets vary from 3–6‰ [27,82,86–88]. Lacking any clear empirical reason to favor particular trophic enrichment factor (TEF) values, we consider various combinations of TEF values in a Bayesian modeling framework in which we identify the values that offer the best fit to the data [89]. We discuss this method in more detail below.

Foodweb δ^13^C and δ^15^N values for candidate subsistence resources such as C_3_ plants, C_4_ plants (maize), large mammals, and freshwater fish were compiled from published data [25,49,54,75,77–79,90]. This sample expands on previous baseline food data for the Andean Altiplano [79,90], and thus allows us to limit our baseline to samples from this territory to avoid altitudinal and other regional effects [91]. For modern samples, δ^13^C values are corrected by +1.5‰ for Suess effects for comparison with ancient samples [91]. To account for meat bone offset, bone collagen samples with δ^13^C are adjusted by −2.4‰ for terrestrial samples [50] and a −3.7‰ offset if the samples are aquatic [27,92]. The δ^13^C and δ^15^N values for Archaic populations predating Kaillachuro and Jiskairumoko were compiled from published data on the Soro Mik’aya Patjxa and Wilamaya Patjxa sites [27]. These values serve as a reference for assessing the subsistence patterns of early foragers in comparison to those expected for early farmers. Wilamaya Patjxa (ca. 9.0 cal. ka) and Soro Mik’aya Patjxa (8.0–6.5 cal. ka) are open-air archaeological sites located in the Titicaca Basin, primarily used for funerary purposes [27,36,93,94]. These sites offer valuable insights into the lifeways of early forager societies prior to the adoption of agriculture in the region.

### Statistical analysis

Basic statistical analysis is performed using R statical computing environment [74], including the creation of a bivariate plot of δ^15^N and δ^13^C values for the human consumer and food-source data. Food resources are displayed as 95% quantile ellipses. We also compute summary statistics for each group in the dataset. Boxplots are used to visually explore the distribution of consumers’ raw values over time (S3 Data in S1 File). Rather than using traditional archaeological periods, we grouped the data into three chronological blocks based on calibrated radiocarbon dates: 9.0–6.5 cal ka (WMP and SMP), 5.3–4.0 cal ka, and 4.0–3.0 cal ka (KCO and JKM). Linear regression models are used to evaluate the predicted decline in δ^13^C and δ^15^N values over time, which is based on median age estimate for each sample. Fitted values and 95% confidence intervals are visualized for each isotopic dataset across periods.

We assessed changes in isotopic variance (δ^13^C and δ^15^N) between chronological groups using both F-tests and Levene’s tests. The F-test evaluates differences in variance assuming normality, while Levene’s test provides a robust alternative less sensitive to outliers. Together, these tests allow us to identify shifts in inter-individual dietary variability across time.

As a related line of evidence for evaluating the relative contributions of meat and plants in consumer diets, isotopic bioapatite values are compared to three regression lines of protein sources following the carbon isotope model outlined by Kellner and Schoeninger [95]. This analysis offers a related line of evidence for evaluating the relative contributions of meat and plants in consumer diets. Data from Andean forager and agricultural populations [25,20] suggest that the values for foraging populations tend to align closely with the regression line representing diets rich in C_3_ protein and C_3_ energy, whether it is a C_3_ plant, or an animal that consumes C_3_ plants. By contrast, maize farming societies are expected to cluster near the C_4_ line. We note that identifying societies that consume C_3_ cultivated plants presents a challenge, as they may be indistinguishable from foragers who gather these plant types.

We estimate the caloric contribution of each potential food source to the diet of these highland consumers using Bayesian tracer mixing models through the R package MixSIAR [96]. These estimates are based on trophic δ^15^N and δ^13^C values of the consumers and prospective food resources, including C_3_ plants, C_4_ plants, camelids, and freshwater fish. To identify appropriate trophic enrichment factors, which can vary in time and place, we follow Chen et al [27] in comparing Akaike information criterion (AIC) and leave-one-out cross validation (LOOic) values for sixteen models, each using different combinations of carbon and nitrogen trophic enrichment factors within the range of uncertainty [96]. To mitigate the influence of weaning effects on the results, non-adults are omitted from the model.

### Zoarchaeological analysis

Zoarchaeological samples recovered from the open-air site of Kaillachuro originate from features such as human burial contexts, burn pits, offering pits, and midden deposits [41]. Analysis of the faunal assemblage from Kaillachuro, was conducted using the Collasuyo Archaeological Research Institute zooarchaeological comparative collection and various references [97,98]. A faunal specimen is a single bone, tooth, or fragment thereof found in an archaeological context, whereas an element refers to a complete, unmodified, bone or tooth in the skeleton of a particular animal [99]. Taxon, element, portion, side, bone fusion or tooth eruption/wear were recorded for each specimen. Taxonomic abundance is measured by the number of identified specimens (NISP) per taxon (e.g., genus, family, or higher taxonomic category), and also by the minimum number of individuals (MNI), which is a *derived* measure of NISP. To determine MNI, we examined the number of overlapping skeletal parts and considered the “age” of the element (e.g., bone fusion, tooth eruption). While these quantitative units are commonplace in zooarchaeological research, it is important to recognize they are not definitive calculations. Gifford-Gonzalez [100] discusses how NISP can be biased by variability in the number of elements in taxa, butchery/transport, different human breakage strategies, bone durability, and collection biases. The statistical validity of tests using NISP is furthermore compromised by the fact that each bone fragment may not come from the same individual [101]. Although MNI addresses some of the statistical constraints on NISP, MNI can present other complications depending on how researchers implement it. As it is derived from NISP, MNI is highly influenced by sample size, making intersite comparisons misleading [101]. Another issue is the aggregate effect, which occurs when differences in MNI arise when it is derived from the whole site assemblage versus stratigraphic units [101]. Due to the complicated stratigraphic sequence of Kaillachuro site, as the result of reburial events [41,75], analysis was performed on the full site assemblage of faunal bones, rather than on individual strata.

Specimens with suitable landmarks were measured using a digital caliper according to standards [102]. The taxonomic profile of the Kaillachuro assemblage is compared to that of the contemporaneous site of Jiskairumoko [22], and the predecessor sites of Soro Mik’aya Patjxa and Wilamaya Patjxa [27] in order to evaluate the expectation of dietary expansion with increasing prominence of low-ranked resources during the period of economic transition. Comparative samples come from the features of these sites that include burial pits, possible roasting or storage pits, and, in the case of Jiskairumoko, pit houses [22].

Animal-bone assemblages are the product of several agencies, including: human decision-making for hunting, butchering, and transport, as well as non-human actions of carnivores, scavengers and environmental actors [103,104]. To understand these taphonomic agents [103,105,106], we recorded the type and orientation of bone breakage (e.g., spiral fracture) and percussion marks; the type, placement, and orientation of cut marks; and the type and extent of animal gnawing. We recorded the degree of specimen burning as stages, wherein stage 1 is unburned, stage 3 is fully carbonized, and stage 6 is fully calcined [107]. To assess the degree of breakage across the assemblage, specimens that could not be identified to a skeletal element (e.g., long bone, axial, unidentified) and identified as medium mammal, large mammal, mammal indeterminate, and vertebrate were assigned to size classes: *1 =* *< 1 cm, 2* *=* *1 − 2 cm, 3* *=* *2 − 5 cm, 4* *=* *5 − 10 cm, 5 =* *> 10 cm*. The possible taxa represented in the large size class (camelid, cervid artiodactyl) can weigh between 35–140 kg, 8–12 kg for medium size class (chilla, clupeo, fox, dog), and 900-1100g for the small taxa (rodent, cuy, bird).

### Archaeobotanical analysis

Archaeobotanical observations are used to evaluate the expected diet-breadth expansion. The working model anticipates the increasing prevalence of plant taxa over time, particularly chenopods and potatoes, which are thought to have been domesticated in the region. While recognizing the analytical potential of archaeobotanical remains, we also recognize some of the challenges. The preservation of carbonized seeds in the Andean Altiplano is influenced by oxygen and humidity. Low humidity and reduced oxygen promote long-term survival. Carbonization halts decomposition, preserving plant structures based on their physicochemical properties. Seeds with resistant pericarps are more likely to survive, while fragile tissues may degrade completely [108–111]. In the high Andes, archaeobotanists have identified preservation problems with archaeobotanical remains, which is why they consider it important to distinguish between carbonized and non-carbonized remains [27].

We recovered macrobotanical remains from the flotation of soil sediments, which were subsequently selected for analysis. These remains originate from well-dated archaeological features and deposits at the Kaillachuro site, including Mound 4 and two non-mound areas (Units 3 and 7), spanning the Terminal Archaic to Early Formative periods. The samples were identified using an AmScope LED-144S stereoscope with a magnification of 10 to 40X in the Department of Ethnobotany and Botany of the UNMSM Natural History Museum (Lima-Peru). Taxonomic determination was based on comparative morphometry [26,29,31,112–115], as well as of comparisons to virtual herbaria pages (International Plant Name Index, The Plant List, Tropics) seeds in the UNMSM Herbarium, and a reference catalog of modern specimens made by the authors. The macrobotanical remains were classified into different plant anatomical structures, such as inflorescence, seed, stem, leaf, root, etc.

## Results

Isotopic values from the 16 Kaillachuro and Jiskairumoko individuals and dietary reconstructions are statistically indistinguishable from those of earlier forager populations in the region. Similarly departing from expectations, the taxonomic profile of the faunal assemblage is indistinguishable from that of earlier populations, showing consistent preference for large mammals and only trace amounts of small mammals, birds, and fish across the forager-farmer transition. Finally, the archaeobotanical analysis shows charred chenopods seeds to be the most abundant samples, consistent with the expectation of intensified use of chenopods. Here, we detail these findings and their articulation with model expectations.

### Stable isotope analysis

**Fig 2:**
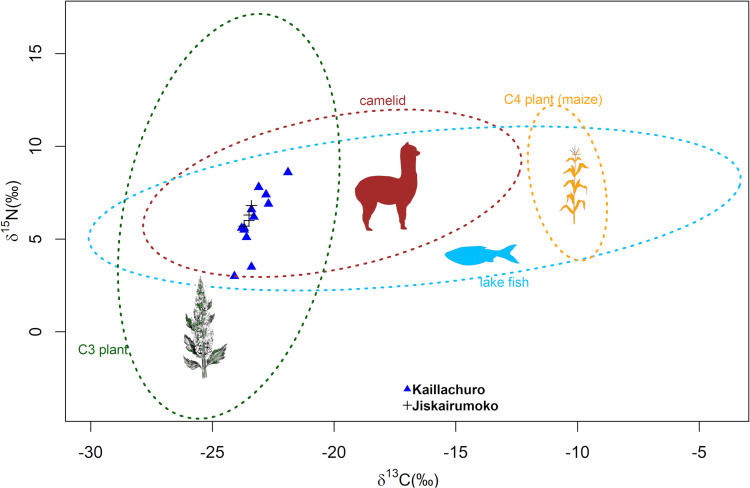
Stable isotope chemistry of human bone samples from Kaillachuro and Jiskairumoko indicate a plant-dominant diet. δ^13^C and δ^15^N values biplots for the consumers and food sources (S1 Table in S1 File). Triangles = Kaillachuro samples, Crosses = Jiskairumoko samples, Ellipses = 95% quantile ranges for each food category.

Our comparative dataset results in the compilation 210 published food web isotopic values from archaeological (67%) and corrected modern (33%) samples from the Andean Altiplano [25,27,49,54,75,77–79,90] (S1 Table in S1 File). There are 20 humans, 47 camelids, 120 C_3_ (domesticated and wild) plants, 6 C_4_ plants (maize), and 17 freshwater fish samples from lakes Titicaca and Uru Uru, Peru and Bolivia. The carbon and nitrogen values in the data strongly cluster by category, indicating a meaningful baseline for comparison with the new human bone samples reported here (Fig 2).

**Fig 3:**
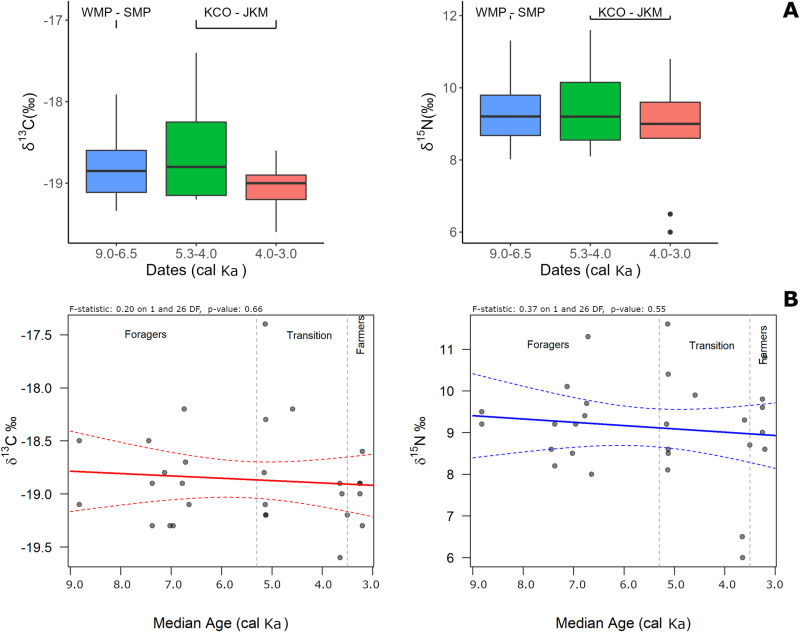
Diachronic analysis of diet in the Titicaca Basin. **A)**: δ ^13^C (left) and δ ^15^N (right) isotope values distribution across three chronological time frames: 9.0–6.5 ka (20 samples from WMP-SMP), 5.3–4.0 ka (9 samples from KCO-JKM), and 4.0–3.0 ka (7 samples from KCO-JKM) that roughly correspond to the Early—Late Archaic, Terminal Archaic, and Terminal Archaic—Early Formative periods, respectively. **B)** Regressions on the continuous data fail to detect any statistical change over the 6000-year time transect (9.0-3.0 cal ka).

Stable isotope characterization was accomplished for 16 individuals from Kaillachuro (n = 12) and Jiskairumoko (n = 4). Atomic C/N ratios of all samples range between 3.1–3.5, within the acceptable range of 2.9–3.6 defined by DeNiro [116]. This suggests that diagenetic processes have not substantially impacted collagen isotopic values. Individuals exhibit dietary δ^15^N_AIR_ values ranging from 6.0–11.6‰, with a mean of 9.0 ± 1.4‰, and dietary δ^13^C_VPDB_ values ranging from −19.6‰ to −17.4‰, with a mean of −18.9 ± 0.5‰ (Figs 2 and 3). These mean values are statistically indistinguishable from the mean δ^15^N value of 9.4‰ and mean δ^13^C value of −18.7‰ observed at the nearby Middle and Late Archaic period individuals in the same region [27]. We therefore fail to find support for the predicted decrease in δ^15^N and δ^13^C values during the period of earliest food production.

An F-test comparing δ^13^C variance between individuals from 9.0–6.5 ka (early foragers), 5.3–4.0 ka (Terminal Archaic), and 4.0–3.0 ka (transition to Early Formative) reveals a significant increase in inter-individual variability (*p* = 0.05 and *p* = 0.03). In contrast, δ¹⁵N variance remains relatively stable between these first two periods but increases significantly during the transition to the Early Formative (4.0–3.0 ka), as indicated by an F-test (*p* = 0.02). These findings suggest that dietary diversification, particularly in nitrogen sources, became more pronounced during the Early Formative transition. In contrast, Levene’s tests for both isotopes show trends toward increased variability, though neither reaches conventional statistical significance (Table 1).

**Table 1:** F-test and Levene’s test results comparing isotopic variance across temporal groups.

	δ13C				δ15N			
	F-Test		Levene’s test		F-Test		Levene’s test	
Temporal groups	p-value	F	F value	Pr(>F)	p-value	F	F value	Pr(>F)
9.0-6.5 ka vs. 5.3–4.0 ka	**0.05**	3.25	3.14	0.09	0.13	2.45	1.96	0.17
5.3-4.0 ka vs. 4.0–3.0 ka	**0.03**	0.18	3.76	0.07	0.61	1.54	0.06	0.81
9.0-6.5 ka vs. 4.0–3.0 ka	0.46	0.60	0.96	0.34	**0.02**	3.79	2.61	0.12

Human dietary δ^13^C and δ^15^N values fall closest to the mean of C_3_ plants, suggesting diets dominated by C_3_ plants with lesser contributions from other food resources (see Fig 2). Diachronic analysis fails to detect any significant shifts in average isotopic values over time (Fig 3A) or associated with the transition from foraging to early food production (δ^13^C *p* = 0.66 and δ¹^5^N *p* = 0.55, see Fig 3B). Average δ^13^C and δ^15^N values remain stable across the forager-farmer transition and the entire time transect of 9.0–3.0 cal. ka (Fig 3B). Overall, the isotopic data do not support a dietary shift through time, and observed variation likely reflects individual or contextual differences within time periods rather than broad subsistence change.

**Fig 4:**
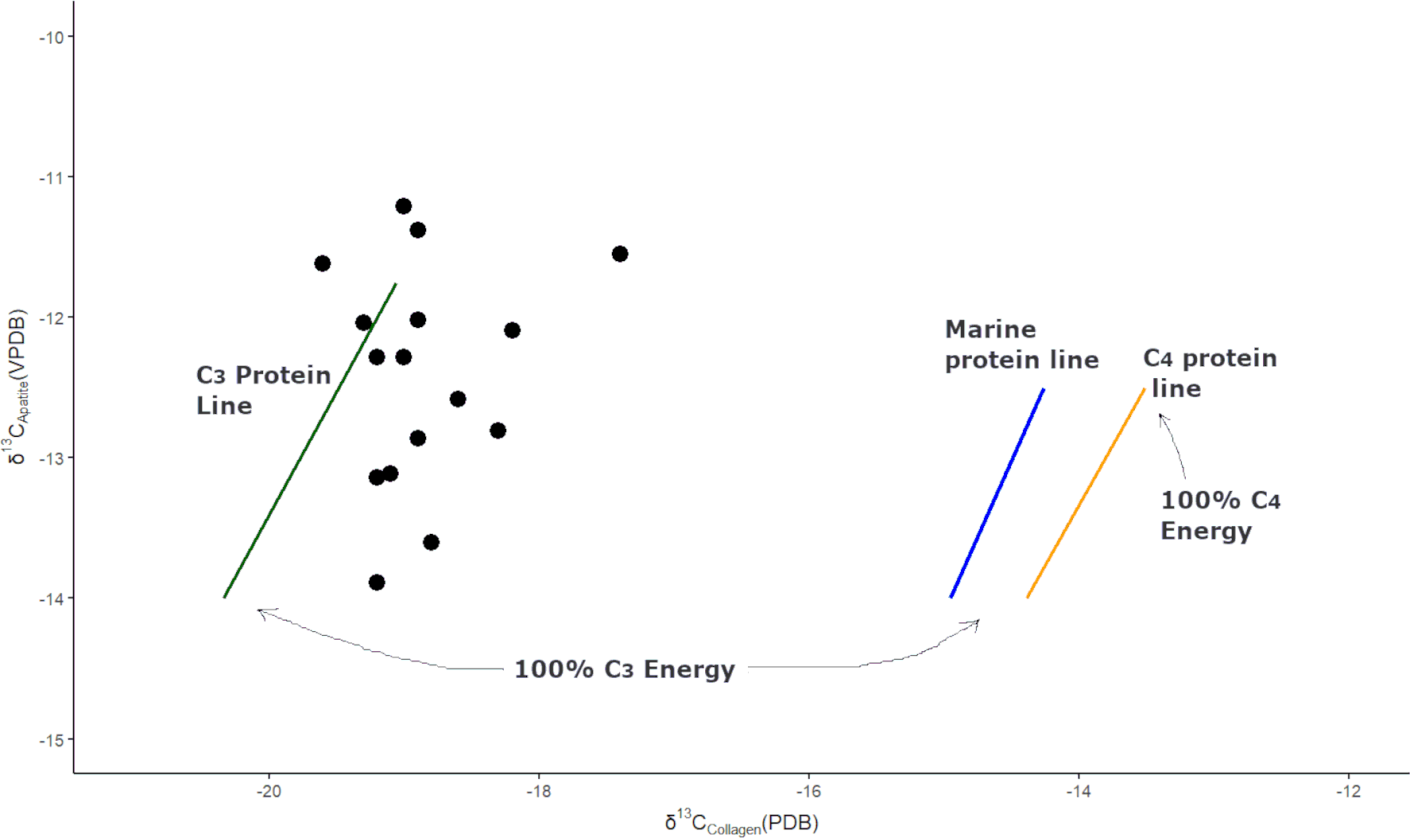
Relationship between δ^13^C_apatite_ and δ^13^C_colagen_ for 16 Kaillachuro and Jiskairumoko individuals revealing C_3_ plant-derived protein. The plot uses three regression lines derived from the Kellner and Schoeninger (2007) model. The regression lines in this model denote the source of protein (C_3_, marine, and C_4_), while the values’ positions (black dots) along the lines indicate the relative proportion of energy (i.e., fats and carbohydrates) provided by the source.

Dietary protein analysis comparing collagen and bioapatite analysis generates results that are consistent with our collagen-based results. δ^13^C_apatite_ and δ^13^C_colagen_ values (Fig 4) show that all individuals are in close proximity to the C_3_ protein regression line, with some even at the base of this line (i.e., 100% C_3_ energy) There are no consumers associated with the other regression lines, such as the marine protein and C_4_ plant lines, which further supports the hypothesis that the Kaillachuro and Jiskairumoko individuals primarily consumed C_3_ plants or terrestrial animals that consumed these plants, such as camelids.

Comparison of Bayesian mixture models under various combinations of trophic-enrichment factors to account for enrichment uncertainty shows that a ^15^N trophic enrichment factor of 3.0‰ and ^13^C trophic enrichment factor of 4.5‰ produce the best fit to the data (Table 2 ). We therefore use these TEF values in our reconstruction of diets while noting that other TEF values produce very similar results.

**Table 2:** Collagen and bioapatite isotope values of human bone from the Kaillachuro and Jiskairumoko sites. The values in the columns δ^13^Cdiet and δ^15^Ndiet are the adjusted isotopic values with trophic enrichment factors of 4.5 for δ^13^C and 3.0 for δ^15^N (more details in S2 Table in S1 File).

ID	Localization	Isotopic data									
	Site	date (95% cal. ka)	Age	Hard tissue types	Sample type	δ^13^C_VPDB_ (‰)	δ^15^N_Air_ (‰)	C:N	δ^13^C_diet_ (‰)	δ^15^N_diet_ (‰)	δ^13^C_apatite_ (‰)
1	Kaillachuro	5.2-5.1	A^b^	Dentin	M3	−19.2	8.6	3.2	−23.7	5.6	−13.9
2	Kaillachuro	5.2-5.1	A	Bone	femur	−19.2	8.5	3.2	−23.7	5.5	−13.1
3	Kaillachuro	5.3-5.0	A	Bone	femur	−19.1	8.1	3.3	−23.6	5.1	−13.1
4	Kaillachuro	5.3-5.0	A	Dentin	LM3	−17.4	11.6	3.2	−21.9	8.6	−11.6
5	Kaillachuro	5.2-5.1	A	Bone	tibia	−18.3	10.4	3.1	−22.8	7.4	−12.8
6	Kaillachuro	ca. 5.3–5.0	A	Dentin	RM2 upper	−18.8	9.2	3.3	−23.3	6.2	−13.6
7	Kaillachuro	ca. 3.8–3.5	A	Dentin	M3	−18.9	6.5	3.3	−23.4	3.5	−12.9
8	Kaillachuro	3.7-3.6	A	Dentin	M3	−19.6	6	3.5	−24.1	3	−11.6
9	Kaillachuro	4.7-4.5	A	Dentin	M3 (B3-UE6)	−18.2	9.9	3.1	−22.7	6.9	−12.1
10	Jiskairumoko	ca. 3.5−3.0^a^	A	Dentin	LM3	−18.9	9.8	3.2	−23.4	6.8	−11.4
11	Jiskairumoko	ca. 3.8–3.2	A	Bone	Bone	−19.2	8.7	3.1	−23.7	5.7	−12.3
12	Jiskairumoko	3.8-3.4	A	Dentin	RM3	−19.0	9.3	3.1	−23.5	6.3	−12.3
13	Jiskairumoko	ca. 3.5–3.0	A	Dentin	LM3	−19.0	9.0	3.2	−23.5	6.0	−11.2
14	Kaillachuro	3.3-3.1	A	Dentin	RI1 lower	−18.6	10.8	3.3	−23.1	7.8	−12.6
15	Kaillachuro	3.3-3.1	A	Bone	femur	−19.3	8.6	3.3	−23.8	5.6	−12.0
16	Kaillachuro	ca. 3.5–3.0	A	Dentin	RM3	−18.9	9.6	3.3	−23.4	6.6	−12.0
mean						−18.9	9.0	3.2	−23.4	6.0	−12.4
SD						0.5	1.4	0.1	0.5	1.4	0.8

^a^ca. = approximate date.

^b^A = Adult.

**Fig 5:**
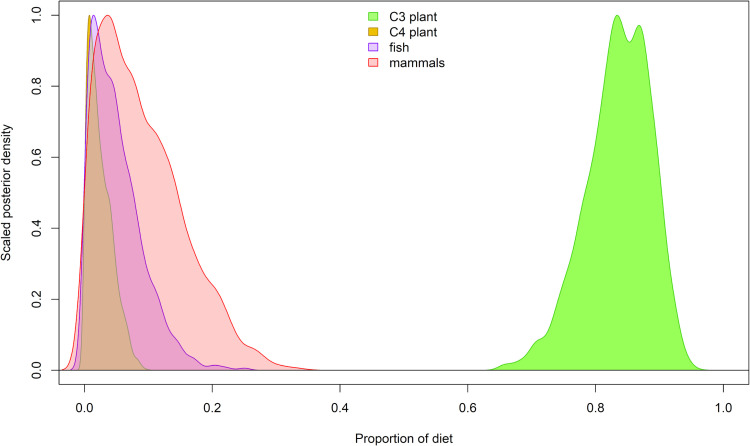
In the subsistence economies of Kaillachuro and Jiskairumoko, the Bayesian mixing models results for the best-fit models indicate that C_3_ plants constituted most of the diet (72-92%), while the remaining sources (mammals, fish, and C_4_ plants) played a secondary role.

The best-fit Bayesian mixture model indicates that C_3_ plants comprised approximately 84% (72–92%) of the average adult diet. Meat and fish comprised only 8% (0–24%) and 4% (0–15%), respectively, while C_4_ plants comprised just 2% (0–7%) (Fig 5, Table 3). The results show that plant foods contributed the majority of calories to individual diets with meat occupying a very distant secondary position. These values indicate that the quantity of C_3_ plant consumption was nearly identical to earlier forager populations of the region, among whom C_3_ plants contributed approximately 84% (73–92%) to the diet [27]. We therefore fail to find support for the prediction of elevated plant consumption in the Terminal Archaic and Early Formative periods, though a high degree of uncertainty in mixing model clouds interpretation. Nonetheless, the consistency in raw isotope values across the Archaic periods favors an interpretation of no dietary change in terms of relative contributions of these broad food categories (see Fig 3).

### Zooarchaeological results

Excavations at Kaillachuro produced a total of 1,978 animal-bone specimens (Table 4). Of these, 17.5% were identified as mammal, 2.6% as birds, and the remainder could only be identified as vertebrate (i.e., bird or mammal; 79.9%). Of the specimens identified to at least order level (n = 94), 92.6% are order Artiodactyla. Camelids comprise a much higher portion (84.6%) of the identified artiodactyls than cervids (15.4%). Just over 5% of the identified assemblage is family Canidae, and 2% is *cuy* (*Cavia* sp.). Large mammals thus comprise 85% and small taxa 15% of identifiable specimens. The taxonomic profile is similar to those of the contemporaneous site of Jiskairumoko and the earlier sites of Soro Mik’aya Patjxa and Wilamaya Patjxa with two exceptions (Table 5). First, the Kaillachuro assemblage has a high proportion of small vertebrate animals compared to the other three assemblages. At first glance, this would seem to support the prediction of diet-breadth expansion. However, closer inspection shows that the Kaillachuro small-taxa component is inflated by a single excavation unit that encountered a special context that contained high frequencies of bird bones, which we describe below. Excluding this observation, all assemblages show similar trace proportions of small taxa and thus fail to support the predicted dietary expansion in the Terminal Archaic Period.

**Table 3:** Comparison of Bayesian mixing models using various trophic enrichment factors. Model 1, with δ^13^C TEF of 4.5 and δ^15^N TEF of 3, has the lowest LOOic and the highest weight, suggesting the best model.

Model	TEF		Sources				Gelman	Geweke	LOOic	weight
**δ** ^**13**^ **C**	**δ** ^**15**^ **N**	**C**_**3**_ **plant**	**C**_**4**_ **plant**	**mammal**	**fish**
1	4.5	3	84(72-92)	2(0-7)	8(0-24)	4(0-15)	0,0,0	1,0,0	1.7	0.328
2	5	3	90(81-96)	1(0-4)	4(0-16)	2(0-10)	0,0,0	8,0,0	1.9	0.297
3	5.5	3	95(88-99)	1(0-3)	2(0-10)	1(0-6)	0,0,0	0,2,2	2.4	0.231
4	6	3	97(90-99)	0(0-2)	1(0-7)	1(0-4)	2,0,0	0,0,0	8.1	0.013
5	4.5	4	85(75-92)	2(0-7)	6(0-21)	5(0-15)	0,0,0	3,1,5	6.5	0.03
6	5	4	91(82-96)	1(0-4)	4(0-14)	3(0-9)	0,0,0	0,0,2	5.4	0.052
7	5.5	4	95(89-99)	1(0-3)	2(0-9)	1(0-6)	2,0,0	0,0,0	5.8	0.042
8	6	4	97(91-99)	0(0-2)	1(0-6)	1(0-4)	1,0,0	0,0,1	10.4	0.004
9	4.5	5	86(74-92)	2(0-7)	6(0-21)	5(0-15)	1,0,0	0,3,0	16	0
10	5	5	92(84-97)	1(0-4)	3(0-13)	2(0-9)	0,0,0	2,7,1	14.2	0.001
11	5.5	5	96(89-99)	1(0-3)	2(0-8)	1(0-6)	0,0,0	1,0,0	14	0.001
12	6	5	97(92-99)	0(0-2)	1(0-6)	1(0-4)	1,0,0	3,4,1	18.6	0
13	4.5	6	85(74-92)	2(0-7)	6(0-22)	5(0-15)	1,0,0	4,0,0	27	0
14	5	6	91(83-96)	1(0-4)	4(0-14)	2(0-9)	1,0,0	0,4,3	24.9	0
15	5.5	6	95(88-99)	1(0-3)	2(0-9)	1(0-6)	2,0,0	0,1,1	25.5	0
16	6	6	97(92-99)	0(0-2)	1(0-6)	1(0-4)	1,0,0	4,0,0	29.9	0

**Table 4:** Number of identified specimens (NISP) and minimum number of individuals (MNI) from the Kaillachuro faunal assemblage.

TAXA	NISP	MNI
Bird (indet.)	52	1
Cuy (*Cavia* sp.)	2	1
Canid (chilla, clupeo, fox, dog)	5	1
Camelid (alpaca, llama, guanaco, vicuña)	33	3
Cervid (huemul, taruca, white-tailed deer)	6	2
Artiodactyl	48	n/a
Large mammal	2	n/a
Medium mammal	60	n/a
Mammal (indet.)	190	n/a
Vertebrate (indet.)	1580	n/a
**Total**	**1978**	**7**

A second domain of variance is observed in the camelid:deer ratios (see Table 5). Whereas camelids comprise approximately 75% of camelid/deer elements at Soro Mik’aya Patjxa and Willa Maya Patjxa, they comprise 85% at Kaillachuro and 90% at Jiskairumoko. The data support previous findings of increasing representation of camelids relative to deer during the Terminal Archaic Period, consistent with a model of animal management beginning during that time if not earlier [22].

Spiral or fresh fractures were identified on 23% and cut marks were observed on 19.5% of the artiodactyl remains. Slightly more than 33% each of the specimens identified as either camelid or cervid had cut marks, which included shallow cuts, deep hack or chop marks, and grooving/sawing marks. Most of the marks are associated with disarticulation at the joints (e.g., acetabulum, patella, femur head) and removal of the lower limbs (e.g., astragalus, ends of phalanges). Nearly every bone in the Kaillachuro assemblage is burned to some degree (99%), although only 2.4% of the specimens were partially calcined and none were entirely calcined, which is more common when bone is used for fuel and/or placed in a hearth for an extended period (Lyman 1994). Most of the bones were either half charred or fully carbonized (80.6%). As bone is burned it becomes more friable, and other taphonomic processes such as trampling and sediment pressure/movement reduce the size of bone fragments. All bird bones are < 1 cm in size. Vertebrate specimens are either <1 cm (83.7%) or 1–2 cm in size (16.3%), and the mammal fragments are similarly patterned. It seems likely that a significant portion of these unidentified mammal and vertebrate pieces are also remains of camelids and cervids. Unfortunately, the extent of burning will likely inhibit further identification of these small fragments, for example, by using ZooMS [117]. Nonetheless, the high degree of burning of faunal remains—which contrasts with the human bone that is almost entirely unburnt— coupled with the presence of cut-marked specimens, indicates cultural consumption of the animal taxa.

The majority of the faunal remains originate from well-dated archaeological deposits [41,75], alongside others coming from features that require explanation to contextualize the proportions of our findings more effectively. The high proportion of bird remains identified at Kaillachuro is limited to a single archaeological context within a hearth dated to the Early Formative occupation of Mound 6. In addition to this context, we discovered at least one burn pit containing camelid remains at Mound 4 of Kaillachuro, which dates to the Terminal Archaic Period. Jiskairumoko also contains pit ovens containing faunal remains [39].

### Archaeobotanical results

Favorable preservation of the anatomical structures of the botanical remains allowed their taxonomic determination. The most common plant food resources in the Kaillachuro archaeobotanical assemblage are 23 burned seeds determined to be from the Chenopodiaceae family (Table 6), most of which come from stratum #3 of Mound 4, which dates to the Terminal Archaic Period [41]. Phenotypic traits of these seeds, alongside experimental investigations of contemporary quinoa specimens, indicates that the burned condition of these plants was likely due to human cooking practices. The rest of the materials correspond to unburned archaeobotanical remains; they are mainly non-food resources, such as grass (Fam. Poaceae) and other herbs (Fam. Lamiaceae). We found a few (n = 7) true potato seeds (TPS) of the Fam. Solanaceae [118,119] in the Terminal Archaic and Early Formative deposits of Mound 4, as well as in the fill of Burial 5. However, none are burned, suggesting modern intrusions.

**Table 5:** Comparison of Kaillachuro faunal profile to that of the contemporary site of Kaillachuro and the earlier sites of Soro Mik’aya Patjxa and Wilamaya Patjxa [22].

Size class	Taxa	Kaillachuro NISP	Percent Large	Jiskairumoko	Percent Large	Soro Mik’aya Patjxa	Percent Large	Wilamaya Patjxa	Percent Large
Large	Artiodactyl	48	32.20%	143	1.01%	108	3.02%	68	11.07%
	Camelid	33	22.10%	576	4.07%	54	1.51%	27	4.40%
	Cervid	6	4.01%	38	0.27%	17	0.48%	3	0.49%
	Mammal Large	2	1.34%	13032	92.20%	3198	89.53%	462	75.24%
	Mammal Large/Medium	60	40.30%	323	2.28%	173	4.84%	32	5.21%
	Camelid (c.f.)	0	0%	21	0.15%	16	0.45%	12	1.95%
	Cervid (c.f.)	0	0%	5	0.04%	6	0.17%	10	1.63%
	**Sum**	**149**		**14138**		**3572**		**614**	
			**Percent Small**		**Percent Small**		**Percent Small**		**Percent Small**
Small	Carnivore	5	8.50%	1	1.32%	1	2.70%	0	0%
	Bird	52	88.10%	3	3.95%	4	10.81%	0	0%
	Fish Indeterminate	0	0%	3	3.95%	3	8.10%	0	0%
	Mammal Medium	0	0%	2	2.63%	3	8.11%	0	0%
	Mammal Small	0	0%	67	88.16%	22	59.46%	1	100%
	Rodent	2	3.40%	0	0%	4	10.81%	0	0%
	**Sum**	**59**		**76**		**37**		**1**	
									
**Grand total**		208		14214		3609		615	
**Proportion large**		71.6		99.5		99.0		99.8	
**Proportion small**		28.4		0.5		1		0.2	

Comparison with archaeobotanical remains at Soro Mik’aya Patjxa [27] reveals an apparent differential emphasis on chenopodium seeds and tubers. Whereas chenopodium seeds are common in the Kaillachuro assemblages, they are rare in the Soro Mik’aya Patjxa assemblage. Conversely, whereas burnt parenchyma tissue is absent in the Kaillachuro assemblage, it is common in the Soro Mik’aya Patjxa assemblage. While the assemblages are small and limited, they provisionally suggest a shift in emphasis from tubers in the Middle and Late Archaic periods to chenopodium seeds in the Terminal Archaic Period. However, previous starch grain analysis from the nearby Terminal Archaic Period site of Jiskairumoko suggests that tubers were present during the Terminal Archaic [23]. Therefore, the absence of archaeological evidence of tubers at Kaillachuro should be interpreted with caution. This may be due to several factors, including preservation issues, the small size of charcoal fragments recovered through flotation—which complicates identification—and the fact that the analysis of botanical remains is still preliminary. While the presence of tubers cannot be ruled out in future studies, current evidence does not clearly indicate their prominence in the archaeobotanical record at Kaillachuro. These data converge to suggest that tuber use was important across the Archaic, with chenopodium gaining prominence during the Terminal Archaic. These observations—particularly the expansion the Terminal Archaic diet to include chenopodium—are broadly consistent with a model of plant intensification during the Terminal Archaic Period.

**Table 6:** Carbonized and uncarbonized macrobotanical materials from Kaillachuro.

Archaeological context			Period	State	Human food				Other plant					
Block/Mound	SU	Feature			*Chenopodium* sp.		*Solanum* sp.		Poaceae		Lamiaceae		Fabaceae	
					n	organ	N	organ	n	organ	n	organ	n	organ
Unit 3	4		TA	Burned	1	seed	0		0		0		0	
7/non-mound	4		TA		2	seed	0		0		0		0	
M4	6		TA		1	seed	0		0		0		0	
M4	3		TA		19	seed	0		0		0		0	
M4	4A		TA	Non-burned	0		2	seed (TPS)	0		0		0	
M4	4B		TA		0		2	seed (TPS)	0		0		0	
7/non-mound	3		TA		0		1	seed (TPS)	0		0		0	
M4	13	Burial 5	EF		0		2	seed (TPS)	8	inflorescence	4	flower	0	
Unit 3	4		TA		0		0		2	inflorescence	0		0	
Unit 3	5		TA		0		0		1	inflorescence	0		0	
M4	8		TA		0		0		1	seed	0		1	seed
7/non-mound	4		TA		0		0		1	inflorescence	11	flower	0	
M4	15	Burial 5	EF		0		0		3	inflorescence	0		0	
M4	7		TA		0		0		2	inflorescence	0		0	

M4 = Mound 4, SU = Stratigraphic Unit, TA = Terminal Archaic, EF = Early Formative, TPS = True Potato Seed.

## Discussion

Prevailing views of agricultural origins envision a transitional period of economic hardship in response to population packing and resource stress, which catalyzed diet-breadth expansion and plant-food intensification. Such a model of trophic decline anticipates carbon, and especially nitrogen, isotope decreases in human bone chemistry across the agricultural transition. Contrary to this expectation, the average isotopic composition of early agricultural individuals from the Titicaca Basin reported here are equivalent to those of preceding Archaic foragers [27], with δ¹⁵N and δ^13^C values from both Kaillachuro and Jiskairumoko remaining remarkably consistent through time. The best-fit Bayesian mixing model Kaillachuro and Jiskairumoko values indicate that, like earlier Archaic diets, C_3_ plants dominated (84%) Terminal Archaic and Early Formative diets in the Andean highlands. While it is still unclear whether Kaillachuro and Jiskairumoko populations relied more on domesticated plants or wild plants, archaeobotanical analysis at Kaillachuro, and previous research at Jiskairumoko [23], suggests that quinoa and potatoes were the most common plant foods and were either domesticated or on the path to domestication at that time, although still consuming wild species. Isotope results also indicate that meat played a minor dietary role, with zooarchaeological data showing that camelids were the most common meat source. The unanticipated stability in plant:meat ratios across the transition to food-producing economies requires explanation, as it challenges expectations of progressive dietary change and underscores the continuity of forager-based subsistence strategies among the earliest farmers.

Other lines of evidence suggest archery technology and animal domestication also appeared during the Terminal Archaic Period [22,43]. We consider the possibility that such behaviors allowed incipient farmers to sustain relatively constant levels of meat consumption. The abundance of projectile points in Terminal Archaic and Early Formative sites, including at Kaillachuro and Jiskairumoko, suggests that large-mammal hunting remained important in the Terminal Archaic Period [18]. Archery technology may have had two critical effects that allowed for dietary stability across the economic transition. First, it may have enhanced hunting effectiveness, allowing meat consumption to continue at previous levels despite declining wild-animal populations. Second, archery technology may have enhanced social control and reduced raiding, which may have allowed food-producing economies to emerge and stabilize [43].

Beyond mean values, inter-individual isotopic variability provides key insights into the diversity of subsistence practices during the transition to food production. The significant and constant increase in δ^13^C variance between early foragers (9.0–6.5 ka), an early phase of the Terminal Archaic Period (5.3–4.0 ka) and transition to Early Formative (4.0–3.0 ka) individuals suggests that this period witnessed growing experimentation with cultivated plants. Interestingly, δ¹⁵N variance does not show a comparable shift until the subsequent transition to the Early Formative (4.0–3.0 ka), when nitrogen isotopes become significantly more variable. This pattern implies that while the incorporation of plants into diets began earlier, shifts in protein sources—perhaps involving differential access to domestic versus wild animals—as well as increasing variability in the isotopic composition of cultivated plants themselves, became more pronounced only later. The latter may reflect evolving agricultural practices, including the use of organic fertilizers or expansion of cultivation into diverse ecological niches, both of which can influence plant δ¹⁵N values and, by extension, those of consumers. Moreover, the discrepancy of inter-individual δ^13^C and δ^15^N variance results highlight the sensitivity of the results to distributional assumptions, meaning that while there is some evidence of increased dietary diversification, these findings should be interpreted carefully, particularly when considering small sample size or non-normal data distributions. However, the results point to a potential latent effect that warrants further evaluation as more Formative Period samples become available.

Fertilization mechanism has held a particularly prominent role in the Andes where scholars have proposed co-evolutionary mechanisms in which the dung of managed camelids is used to fertilize managed plants, which feed back into sustaining the camelids [79,120–122]. This co-evolutionary model anticipates an increase in δ^15^N values over time, wherein significant amounts of nitrogen in plants are not derived from atmospheric nitrogen, but from animal byproducts which are already elevated in δ^15^N compared to air. However, our data fails to reveal the predicted increase over time. It thus appears that fertilization practices were not yet in place by the Early Formative Period, at least not in the Ilave Basin. Whittemore’s analysis shows that such practices were, however, in place by the Late Intermediate Period [79]. On the coast of northern Chile, bird guano fertilization in the Azapa Valley began with early agriculture during the Formative Period [122]. Likewise, analysis of camelid faunal remains in the Nasca region on the south-central coast of Peru also shows elevated δ^15^N, suggesting fertilization, by 4200 cal BP in the Terminal Archaic [123]. Explicit analysis of additional human samples and the analysis of faunal bone chemistry would be valuable for identifying when fertilization practices first emerged in the Titicaca Basin.

Recent zooarchaeological data indicates a rise in the use of camelids, along with the onset of their cultural management, throughout the Archaic Period in the Titicaca Basin [22]. However, the significance of camelids in the archaeological record remains unresolved, as our isotopic findings seem to contradict their dietary importance. These unexpected results may be clarified by three interpretations that are not necessarily incompatible with one another. First, animals served as key economic resources beyond food, including as wool and hide producers, pack animals to obtain exotic goods such as obsidian, sources of dung used as fuel [124], and as products of exchange themselves. Second, faunal remains identified from the Kaillachuro burial contexts suggest camelids, and to a lesser extent other animals, were processed, cooked, and consumed at the site in different social contexts. The proportion of camelid meat consumption in Kaillachuro is comparable to that in Jiskairumoko village [22], it may be considered regular household usage; however, the presence of secondary burial pits adjacent to the Jiskairumoko pit-houses, some containing faunal remains, likely camelid bones [39,76], suggests a second possibility, that part of these remains could be leftovers from ceremonial consumption. Furthermore, the presence of at least two features with buried animal bones at Kaillachuro suggests that they were provided to the dead as food offerings. Third, the management of camelid herds may have been incentivized by competitive male signaling [8,125,126]. This could have been a result of competitive herd display and the establishment of interregional exchange networks, which allowed males seeking status to obtain and show off prestigious goods [8,57]. The camelid remains at Kaillachuro and Jiskairumoko thus may represent a mix of both wild and managed camelids, used in complex social contexts involving domestic consumption, exchange, rites, and costly signaling.

A limitation in our analysis is the absence of data between 6.5 and 5.3 cal ka, which leaves a gap in the last part of the forager occupation. However, the forager data from 9.0 to 6.5 cal ka already show a consistent dietary pattern, which closely aligns with that of the transition period (5.3–3.5 cal ka) and first farmers (3.5–3.0 cal ka). While the gap necessitates caution in our interpretation of potential changes during this period, the available data strongly suggest that the dietary strategy remained largely stable through millennia, with early farmers adopting a similar foraging-based diet.

These findings suggest that early farming populations in the region retained a forager-like dietary pattern, even in the presence of domestic crops. Rather than a rapid and uninterrupted shift, the results may show  the gradual, non-linear, and potentially mosaic or arrhythmic nature of subsistence transitions, where cultivated plants—at various stages of domestication—were integrated into existing practices without immediately displacing traditional wild food sources [5,10,68,71,127]. Alternatively, the isotopic stability may point to continued reliance on wild resources for protein, while early unfertilized crops—largely C₃ species (basically potatoes and chenopods)—had minimal isotopic impact. This persistence highlights the adaptive flexibility of early agropastoral communities in the Titicaca Basin as they navigate new economic options.

## Conclusion

Results in this study challenge the current view that the transition to food-producing economies entailed diet-breadth expansion and increased plant-food consumption. Rather, the Andean Altiplano case reveals surprising dietary stability across the transition from foraging to farming economies. Both forager and early farmer diets were primarily composed of C_3_ plants with lesser caloric contributions from large mammals, including camelids and deer. Surprisingly, this subsistence regime was maintained for some four millennia despite human population growth across the Archaic and Formative periods at the Altiplano. Although it is likely that during this transition period in Kaillachuro and Jiskairumoko, there was a gradual and flexible integration of domestic crops into the diets of Archaic foragers, reflecting a mosaic subsistence transition rather than an abrupt shift to agriculture. The Andean case thus represents a remarkable case of economic resilience in the face of demographic and economic transformation. Evidence for expanding trade networks and archery technology during the Terminal Archaic Period suggests that social and technological innovations are the likely explanations for subsistence stability across the forager-farmer transition. This feat of resilience not only allowed Andean Altiplano populations to maintain previously successful dietary regimes but also resulted in the domestication of plants and animals that would go on to fuel the later emergence of urban centers, intensive agricultural strategies, and some of the world’s most expansive socioeconomic systems, including the Tiwanaku and Inca phenomena.

## Supporting information

S1 File **S1 Table. Food Web Data**. 210 published isotopic values from archaeological and adjusted modern samples from the Andean Altiplano. **S2 Table. Isotopic and archaeological context data of human bones**. 16 isotopic values from the Kaillachuro and Jiskairumoko sites. **S3 Data. R code for statistical analysis.**
(ZIP)
